# Enhancement of Host Immune Responses by Oral Vaccination to *Salmonella enterica* serovar Typhimurium Harboring Both FliC and FljB Flagella

**DOI:** 10.1371/journal.pone.0074850

**Published:** 2013-09-17

**Authors:** Jeong Seon Eom, Jin Seok Kim, Jung Im Jang, Bae-Hoon Kim, So Young Yoo, Ji Hyeon Choi, Iel-Soo Bang, In Soo Lee, Yong Keun Park

**Affiliations:** 1 School of Life Sciences and Biotechnology, Korea University, Seoul, Korea; 2 Department of Microbiology and Immunology, Chosun University School of Dentistry, Gwangju, Korea; 3 Department of Biotechnology, Hannam University, Daejeon, Korea; Indian Institute of Science, India

## Abstract

Flagellin, the structural component of the flagellar filament in various motile bacteria, can contribute to the activation of NF-κB and proinflammatory cytokine expression during the innate immune response in host cells. Thus, flagellin proteins represent a particularly attractive target for the development of vaccine candidates. In this study, we investigated the immune response by increasing the flagella number in the *iacP* mutant strain and the adjuvant activity of the flagellin component FljB of *Salmonella enterica* serovar Typhimurium. We found that the *iacP* mutant strain expresses two flagellin proteins (FliC and FljB), which result in increased NF-κB-dependent gene expression in bone marrow derived macrophages. Using an oral immunization mouse model, we observed that the administration of a live attenuated *S* *. typhimurium* BRD509 strain expressing the FliC and FljB flagellins induced significantly enhanced flagellin-specific IgG responses in the systemic compartment. The mice immunized with the recombinant attenuated *S* *. typhimurium* strain that has two types of flagella were protected from lethal challenge with the *Salmonella* SL1344 strain. These results indicate that overexpression of flagella in the *iacP* mutant strain enhance the induction of an antigen-specific immune responses in macrophage cell, and both the FliC and FljB flagellar filament proteins-producing *S* *. typhimurium* can induce protective immune responses against *salmonellosis*.

## Introduction

*Salmonella enterica* serovar Typhimurium ( *S* *. Typhimurium* ) is a Gram-negative flagellated bacterial pathogen that can cause a variety of diseases in different hosts, including gastroenteritis, typhoid fever, focal infections and septicemia [1,2]. Typhoid fever caused by S. Typhi remains a serious complication with high rates of mortality, being estimated to cause more than 600,000 deaths per year in many developing countries [3]. However, the present vaccine approaches have limited efficacy and safety. Therefore, the development of cost-effective and safe vaccines against *Salmonellosis* remains an important challenge.

*Salmonella* has several pathogen-associated molecular patterns (PAMPs), such as flagellin, lipopolysaccharide (LPS), microbial DNA and lipoproteins, which are recognized by the Toll-like receptor 5 (TLR5), TLR4, TLR9 and TLR2, respectively [4]. Recognition of PAMPs by TLRs initiates a series signaling cascades leading to the activation of the transcription of nuclear factor-κB (NF-κB) and triggers the production of pro-inflammatory cytokines and chemokines that direct the adaptive immune response. The intracellular NOD-like receptors (NLRs) are located in the cytoplasm and can also recognize intracellular PAMPs [5].

Bacterial flagella are appendages on the cell surface that are required for the motility and chemotaxis of bacterial pathogens and for epithelial cell invasion as a virulence factor [6,7]. Flagellins, a key component of the bacterial flagella, are recognized as PAMPs in host cells, through binding with TLR5 and NLRC4. Stimulation of TLR5 by extracellular flagellin induced the secretion of proinflammatory cytokines following NF-κB activation and chemokine production, while flagellin within the cytosol of host macrophages is detected through the NLR during *Salmonella* infection [8,9]. Studies using TLR5-deficient mice have identified that the flagellin component FliC of *S* *. Typhimurium* is capable of activating the innate immune system via a specific interaction with TLR5 to elicit immune protection to *Salmonella* infection [10]. Diphasic *S* *. Typhimurium* possesses two flagellin genes, *fliC* (phase 1 flagellin) and *fljB* (phase 2 flagellin), which are alternatively expressed by Hin recombinase [11]. However, the mechanisms underlying the adjuvant effect of flagellin FliC or other variants of the phase 2 flagellin genes (*fljB*) are currently unresolved.

Recent studies have clearly demonstrated that the bacterial protein flagellin can function as a highly effective adjuvant to exploit the immune response for the development of potent vaccines [12]. Yang et al. observed that the flagellar overexpression attenuates *Salmonella* virulence and elicits an elevated immune response via flagellin adjuvant, thereby conferring robust protection against *Salmonellosis* [8]. We previously found that the expression level of the *fljB* gene was higher in the *iacP* mutant strain grown under SPI1-inducing conditions, although the expression of the *fliC* gene was independent of IacP [7]. As expected, the *iacP* mutant strain produced more flagella than the wild-type strain. In this study, we found that the *iacP* mutant strain simultaneously expressing two flagellin proteins, FliC and FljB, strongly induced NF-κB activation in vitro and *ex vivo*, thereby promoting the host immune response.

Attenuated *S* *. Typhimurium* strains have been investigated intensively as live carriers of heterologous antigens for a potential vaccine approach [13]. We evaluated the effectiveness of immunization with flagellar overexpression in mice following oral inoculation with an attenuated *S* *. Typhimurium* BRD509 strain variant, which was designed to produce FljB and FliC. The immune responses induced by these recombinant attenuated strains expressed high levels of serum anti-LPS and anti-flagellin IgG antibodies with increasing IgG levels during the immunization. Additionally, the mice orally immunized with attenuated *S* *. Typhimurium* BRD509 containing both FljB and FliC flagella were protected against a challenge with a virulent *Salmonella* SL1344 strain. These results suggest that the synthesis of more flagellar proteins in a live attenuated vaccine strain can be an attractive strategy to induce protective immunity.

## Materials and Methods

### Ethics statement

All animal experiments were performed in strict accordance with the recommendations in the Guide for the Care and Use of Laboratory Animals of the National Institutes of Health. The protocols for animal experiments were approved by the Institutional Animal Care and Use Committee (IACUC) of Korea University (Permit Number: KUIACUC-2012-96). All effort was made to avoid wounding animals and to minimize additional stress and suffering of animals. A minimal number of mice were used for the study and the mice were administered under inhalation anesthesia with diethyl ether. After challenge, all mice were monitored twice daily for clinical signs, and mortality for a period of 14 days. The humane endpoint of the challenge experimental was a significant body weight loss (>20% of normal body weight) and impairment of mobility (the inability to reach food and water, ruffled fur and squinted eyes) at the time of challenge inoculation, and acceptable method of euthanasia is cervical dislocation. Humane endpoints in animal experiments were also necessary for mice which survived at the conclusion of the experiment.

### Bacterial strains and growth conditions

The *S* *. Typhimurium* strains and plasmids used in this study are listed in Table 1. The bacterial cell cultures were grown at 37°C in Lysogeny broth (LB) with 0.3 M NaCl for SPI-1 activation. For the growth study, overnight cultures of the *S* *. Typhimurium* strains were diluted 100-fold into 100 ml of fresh LB broth containing 0.3 M NaCl. The growth was monitored hourly at OD_600_ over a 24 h period using a spectrophotometer (Spectronic 20D+; Thermo Spectronic, Rochester, USA). For selection, antibiotics were used at the final concentrations: ampicillin (Ap; 100 µg/ml), chloramphenicol (Cm; 30 µg/ml), kanamycin (Km; 50 µg/ml) and tetracycline (Tc; 20 µg/ml).

**Table 1:** *Salmonella* strains and plasmids used in this work.

**Strain or plasmid**	**Genotype or description**	**Source or reference**
Strains		
UK-1	Wild-type	[37]{Moreno, 2000 #40; Moreno, 2000 #1427}
SL1344	Wild-type	[38]
BRD509	SL1344, *aroA aroD*	[38]
M587	SL1344, *fliGHI*::Tn*10*; Tc^r^	[39]
YKJ035	UK-1, Δ*iacP*	[33]
YKJ231	UK-1, Δ*iacP*, *fljB*::*Km*; Km^r^	[7]
YKJ500	UK-1, *fliC*::*Cm*; Cm^r^	This study
YKJ503	UK-1, Δ*iacP*, *fliC*::*Cm*; Cm^r^	This study
YKJ504	UK-1, Δ*iacP*, *fljB*::*Km*, *fliC*::*Cm*; Km^r^ Cm^r^	This study
YKJ505	UK-1, *fljA*::*Cm*; Cm^r^	This study
YKJ211	UK-1, *hin*::*Km*; Km^r^ (on-orientation; FljB^on^)	This study
YKJ212	UK-1, *hin*::*Km*; Km^r^ (off-orientation; FliC^on^)	This study
YKJ506	BRD509, *hin*::*Km*; Km^r^ (FljB^on^)	This study
YKJ507	BRD509, *hin*::*Km*; Km^r^ (FliC^on^)	This study
YKJ508	BRD509, *hin*::*Km*, *fljA*::*Cm*; Km^r^ Cm^r^ (FljB^on^, FliC^on^)	This study
Plasmids		
pKD46	pSC101. P_BAD_-*gam bet exo*, oriTS; Ap^r^	[14]
pKD3	FRT-*cat*-FRT, *oriR6K*; Ap^r^ Cm^r^	[14]
pKD4	FRT-*aph*-FRT, *oriR6K*; Ap^r^ Km^r^	[14]
pMW118	Low copy number plasmid; Ap^r^	Nippon Gene
pBAD24	Expression plasmid, containing arabinose-inducible promoter P_BAD_; Ap^r^	[40]
pYKJ033	pMW118, p*iacP* ^HA^; Ap^r^	[33]
pYKJ507	pBAD24, P_BAD_-*fljB*; Ap^r^	This study
pYKJ508	pBAD24, P_BAD_-*fliC*; Ap^r^	This study

### Construction of *S* *. Typhimurium* mutant strains

The mutant strains were constructed using the lambda-red recombinase method [14,15] with the appropriate primers listed in Table 2. Briefly, the chloramphenicol resistance (Cm^r^) cassettes of the plasmid pKD3 flanked by Flp recombination target (FRT) sites were amplified with the following primers: *fliC* mut-L and *fliC* mut-R for YKJ500, *fljA* mut-L and *fljA* mut-R for YKJ505. The resulting PCR product was electroporated into *S* *. Typhimurium* harboring the lambda-red recombinase plasmid (pKD46), and the transformants were incubated for 1 h at 37°C and then plated on LB agar plates supplemented with chloramphenicol. Insertions of the Cm^r^ gene were verified by colony PCR and DNA sequencing. To generate the *iacP fliC* double mutant strain (YKJ503), the *fliC*::*Cm* allele from YKJ500 was transduced into the *iacP* mutant strain (YKJ035) by P22-mediated transduction. The YKJ504 strain was generated by transducing the *fliC*::*Cm* allele from YKJ500 into the *iacP fljB* mutant strain (YKJ231).

**Table 2:** Primer sequences used in this study.

**Primers**	**Sequences^*a*^**	**Description**
*hin-A-FRT* ^*b*^	5’-CCGCTCTGCGATTTTTATAGCGCATCAGCCACACGATTTTGTAGGCTGGAGCTGCTTCG	YKJ211, YKJ212
*hin-B-FRT* ^*b*^	5’-CTGGGAGGGCGCCCTCGGGCGATCAACAAACATGAACAGGACATATGAATATCCTCCTTAG	YKJ212
*hin-C-FRT* ^*b*^	5’-TCCTGTTCATGTTTGTTGATCGCCCGAGGGCGCCCTCCCAGCATATGAATATCCTCCTTAG	YKJ211
*fliC* mut-L^*c*^	5’-GATCATGGCACAAGTCATTAATACAAACAGCCTGTCGCTGTTGACGTGTAGGCTGGAGCTGCTTC	YKJ500
*fliC* mut-R^*c*^	5’-ATCAATCGCCGGATTAACGCAGTAAAGAGAGGACGTTTTGCGGAACATATGAATATCCTCCTTAGT	YKJ500
*fljA* mut-L	5’-TTGCTCGTCGAGTTCAATTTCTACGTTTTAATGATATGTAGGCTGGAGCTGCTTC	YKJ505
*fljA* mut-R	5’-GTCAAAACCTGTCCATACTTCATATAGATTTTGATACCATATGAATATCCTCCTTAG	YKJ505
B*fljB*-L	5’-CCGGAATTCACCATGGCACAAGTAATCAACACTAACA	pYKJ507
B*fljB*-R	5’-CCGGCATGCTTAACGTAACAGAGACAGCACGTT	pYKJ507
B*fliC*-L	5’-CCGGAATTCACCATGGCACAAGTCATTAATACAAACA	pYKJ508
B*fliC*-R	5’-CCGGCATGCTTAACGCAGTAAAGAGAGGACG	pYKJ508
IL18–L	5’-TCTGACCTCGACTCCGTCCA	RT-PCR, qPCR
IL18-R	5’-ATCTGCTGAAACAACTGCCG	RT-PCR, qPCR
IL1β–L	5’-TGGAATGTATGGCTGTAAATGA	RT-PCR, qPCR
IL1β-R	5’-AGCGTAGTCCGAAGACGTGA	RT-PCR, qPCR
GAPDH-L	5’-AGAGTTTGATCMTGGCTCAG	RT-PCR, qPCR
GAPDH-R	5’-TACGGYTACCTTGTTACGACTT	RT-PCR, qPCR

^a^Underlined sequences represent restriction enzyme recognition sites.

^b^ Primer sequences are described previously [16].

^c^ Primer sequences are described previously [15].

The YKJ211 and YKJ212 strains that expressed only one type of flagellin (phase-locked) were constructed as described previously [16]. The YKJ506 and YKJ507 strains were generated by P22 transduction of the *hin*::*Km* alleles from the YKJ211 (FljB^on^) or YKJ212 (FliC^on^) into the attenuated *S* *. Typhimurium* BRD509, respectively. To construct the BRD509 *fljB* ^*+*^ *fliC* ^*+*^ (YKJ508), the *fljA*::*Cm* allele from YKJ505 was transduced into the strain YKJ506 by P22-mediated transduction. The phage sensitivity was tested with P22 H5.

### Construction of recombinant plasmids

The plasmid constructs and oligonucleotide primers used in this study are shown in Tables 1 and 2. To construct pYKJ507, the *fljB* gene was amplified from the genomic DNA of the *S* *. Typhimurium* strain UK-1 with primers B*fljB*-L and B*fljB*-R and subsequently inserted into the *EcoR*I/*Sph*I sites of the pBAD24 plasmid. The plasmid pYKJ508 was constructed in the same way as the pYKJ507 construct; the *fliC* gene was amplified from the UK-1 chromosome using primers B*fliC*-L and B*fliC*-R. The PCR product was cloned into the *EcoR*I and *Sph*I restriction sites of the pBAD24 plasmid. All plasmid constructs were verified by DNA sequencing analysis.

### Preparation of the secreted proteins and whole-cell lysates of *S* *. Typhimurium*

For expression of the *fliC* or *fljB* flagellin genes, *S* *. Typhimurium* bacteria were grown under the conditions described above for 3 h. The cultures were then collected by centrifugation at 10,000*g* for 5 min to separate the cell pellet and supernatant. The resulting pellets were directly resuspended in 40 µl of sodium dodecyl sulfate-polyacrylamide gel electrophoresis (SDS-PAGE) sample buffer. For the preparation of the secreted proteins from the *Salmonella* culture, the bacterial supernatant was passed through a 0.45-µm-pore-size syringe filter and then recovered by the addition of trichloroacetic acid (10% vol/vol) and precipitated on ice for 3 h. The resulting protein pellet was washed with ice-cold acetone and resuspended in phosphate-buffered saline (PBS) containing 80 mM Tris-HCl (pH 8.0). Equal amounts of proteins were resolved on 12% SDS-PAGE gels and then subjected to Western blot analysis.

### Western blotting and antibodies

The separated proteins in the gel were transferred onto nitrocellulose membrane by electroblotting (120 V, 1 h) using a Mini Trans-Blot Electrophoretic Transfer Cell (Bio-Rad, CA, USA). The nitrocellulose membranes were blocked with 5% skim milk in TBS-T (Tris-buffered saline with 0.1% Tween-20) for 1 h and then incubated with one of the following primary antibodies at the proper dilution for 1 h: a polyclonal anti-FliC antibody (1:3,000; Becton Dickinson, Franklin Lakes, NJ, USA); a polyclonal anti-FljB antibody (1:5,000; Becton Dickinson, Franklin Lakes, NJ, USA), a monoclonal anti-DnaK antibody (1:5,000; Enzo Life Sciences, Farmingdale, NY, USA), a polyclonal anti-p65 antibody (1:500; Santa Cruz Biotechnology Inc., Santa Cruz, CA), a polyclonal anti-Lamin-B antibody (1:1,000; Santa Cruz Biotechnology Inc., Santa Cruz, CA), a polyclonal anti-GAPDH antibody (1:2,000; Santa Cruz Biotechnology Inc., Santa Cruz, CA) and a polyclonal anti-IL-18 antibody (1:500; Santa Cruz Biotechnology Inc., Santa Cruz, CA). After washing with TBS-T, the membranes were incubated with horseradish peroxidase (HRP)-conjugated goat IgG secondary antibodies (1:3,000; Bio-Rad, Berkeley, CA, USA) for 1 h. The blots were developed using a BM chemiluminescence blotting substrate (POD) (Roche, Mannheim, Germany).

### lmmunogold labeling and transmission electron microscopy (TEM)

The bacteria cultures were centrifuged at 930*g* for 20 min, washed two times with distilled water (DW) and fixed for 10 min with 4% paraformaldehyde. Carbon Formvar-coated copper grids (200-meshes) was rendered hydrophilic by high-voltage glow discharge (JFC- 1100E ION SPUTTER, JEOL Co., Tokyo, Japan) and floated on the fixed suspensions for 3 min. After washing with DW, the grids were blocked in 2% BSA for 1 h, rinsed with DW and incubated with anti-FljB antibody (1:500) as the primary antibody for 30 min at room temperature (RT). After washing three times with DW, 10-nm-gold-labelled anti-rabbit goat IgG (1:3,000; Abcam Inc., USA) diluted in DW was added to the grids and incubated for 30 min at RT [17]. The bacteria on the grids were washed two times with DW, negatively stained with 2% uranyl acetate for 30 sec and subsequently rinsed three times with DW. The samples were examined under a Tecnai 12 TEM (Philips, Eindhoven, Netherlands) at an acceleration voltage of 120 kV.

### Flagella staining

The observation of the flagellar subunits FliC or FljB in *S* *. Typhimurium* bacteria was performed as described by Valentina [18] with some modifications. The bacterial cells were incubated with Hoechst 33342 dye (1:1,000; Invitrogen, CA, USA) for 20 min prior to fixation. A 500 µl volume of bacterial culture was mixed with 100 µl of 16% paraformaldehyde in PBS for 20 min at RT. The fixed bacteria cells were washed three times with PBS for 2 min and then blocked in 2% BSA in PBS for 60 min at RT. To visualize the flagellum, the bacterial cells were treated with anti-FliC antibody (1:200) and anti-FljB antibody (1:500) overnight at 4°C. The samples were washed five times with PBS and subsequently treated with Alexa Fluor 488-conjugated (1:200; Invitrogen, CA, USA) or Alexa Fluor 568-conjugated goat anti-rabbit secondary antibody (1:200; Invitrogen, CA, USA) for 60 min at RT in the dark. The samples were washed five times with PBS and mounted in using Fluoromount-G (Southern Biotech, Birmingham, AL, USA) antifade medium. The specimens were observed with a confocal laser scanning microscope (LSM 700; Carl Zeiss, Germany). Images from three different confocal planes (at least 50 cells) per sample were analyzed to determine the quantification of the number of flagella per cell.

### Isolation of cytosolic and nuclear fractions

Cytoplasmic and nuclear extracts were prepared using a modified version of the method described by Pieper, G [19]. RAW 264.7 cells (Mouse leukaemic monocyte macrophage cell line) and bone marrow derived macrophages (BMDM) were seeded into 100-mm-diameter culture dishes at 5 × 10^6^ cells/dish for 24 h and then infected with bacteria at an MOI (multiplicity of infection) of 50 for 60 min. The cells were washed three times with PBS and harvested by scraping in hypotonic buffer A (10 mM HEPES [pH 7.9], 1.5 mM MgCl_2_, 10 mM KCl, 0.2 mM PMSF, 0.5 mM DTT) to obtain cytosolic and nuclear fractions. After centrifugation of the samples at 3,300*g* for 5 min at 4°C, the supernatants were collected and used as cytoplasmic extracts. The pellets were then resuspended in hypertonic buffer B (20 mM HEPES [pH 7.9], 0.2 mM EDTA, 25% [vol/vol] glycerol, 1.5 mM MgCl_2_, 0.4 M NaCl, 0.5 mM DTT) and incubated on ice for 10 min [20]. After centrifugation at 13,000*g* for 10 min, the supernatants were collected and used as nuclear protein extracts. The total protein concentrations were determined using a Bradford protein assay (Bio-Rad, Berkeley, CA, USA). Equal amounts of cytoplasmic and nuclear proteins were separated on 12% SDS-PAGE gels and subjected to Western blotting.

### Isolation of bone marrow-derived macrophages (BMDMs)

Bone marrow was isolated from 5-week-old female C57BL/6 mice (Labanimal, Seoul, South Korea) killed by cervical dislocation to generate BMDMs using a method for deriving standards [20,21]. Bone marrow was harvested from the femur and tibia, disaggregated, washed and resuspended in DMEM medium supplemented with 30 ng/ml of recombinant mice macrophage colony-stimulating factor (M-CSF; R&D Systems Europe Ltd, Abingdon, UK). Aliquots of this suspension were seeded in each petri dish and incubated at 37°C in a 5% CO_2_ atmosphere for six days. Two days after seeding, an additional 10 ml of fresh M-CSF was added per plate and incubated for an additional four days. On day six, the non-adherent cells were removed by three PBS washes. The washed cells were seeded at 1 × 10^6^ cells/well in 6-well plates and incubated at 37°C overnight. The cells were stimulated with bacteria at an MOI of 50 for 60 min, and then the cytoplasmic and nuclear extracts were prepared by the above method.

### Detection of p65 nuclear translocation

The nuclear translocation of the NF-κB p65 subunit as a hallmark of NF-κB activation was determined as described by Rutledge [21] with some modification. The *S* *. Typhimurium* bacteria were incubated with Hoechst 33342 dye (1:1,000; Invitrogen) for 20 min prior to infection. RAW 264.7 macrophages and BMDMs grown on glass cover slips in 24-well plates were infected with *S* *. Typhimurium* at an MOI of 50 for 45 min. After infection, the macrophages on the coverslips were washed three times with PBS, fixed with 3.7% paraformaldehyde in PBS at RT for 20 min and permeabilized with 0.2% (vol/vol) Triton X-100 in PBS for 10 min. The permeabilized cells were incubated with 3% (wt/vol) BSA in PBS for 60 min at RT to suppress non-specific binding. The cells were incubated with p65 antibody (1:500) overnight at 4°C, followed by incubation with Alexa Fluor 488-conjugated goat anti-rabbit secondary antibody (1:200) in blocking buffer (2% BSA) for 60 min at RT in the dark [20]. Following mounting, the cells were detected using a confocal laser scanning microscope (LSM700; Carl Zeiss).

### RNA isolation, reverse transcription-PCR (RT-PCR) and quantitative real-time PCR (qPCR) analysis

RAW 264.7 macrophages were stimulated for 2 h at 37°C with *S* *. Typhimurium* strains and harvested for total RNA isolation using the RNeasy plus mini kit (Qiagen, Hilden, Germany). The total amount of RNA was determined using a UV/VIS spectrophotometer (Nano-drop, Thermo, ND-1000). First strand cDNA was synthesized using 1 µg of isolated RNA template, M-MLV reverse transcriptase, Oligo(dT) 15 Primer, RNase inhibitor and dNTP according to the manufacturer’s protocol (Promega, Madison, WI, USA). A subset of the genes was amplified with Ex-taq DNA polymerase (Takara Bio Inc., Shiga, Japan) using gene-specific primers listed in Table 2. The amplified products were separated on a 1% agarose gel and visualized by ethidium bromide staining. The qPCR analyses were performed using a LightCycler 480 (Roche Applied Science, Indianapolis, IN, USA) in a total volume of 10 µl containing 5 µl of SYBR-green I mixture (Roche), 200 nM each of the primers listed in Table 2 and 2 µl of sample cDNA. The PCR conditions were as follows: 50°C for 2 min, 95°C for 5 min, followed by 45 cycles of 95°C for 30 s, 55°C for 30 s, and 72°C for 30 s. GAPDH was used as a normalization control.

### Invasion assays

INT407 cell (human embryonic intestinal epithelial cell line, ATCC CCL-6) monolayers grown on 24-well plates at a density of 2 × 10^5^ cells/well were infected with *S* *. Typhimurium* strains at an MOI of 10 for 45 min at 37°C. After washing three times with fresh DMEM, the cells were incubated in DMEM containing gentamycin (100 µg/ml) for 90 min to kill the remaining extracellular bacteria. After the plates were washed three times with PBS at RT, the cells were treated with a 1% Triton X-100 (vol/vol) solution for 15 min at RT, and the lysates were plated onto LB agar plates after serial dilution. The invasion rates were calculated as a percentage of the wild-type, which was set at 100%.

### Mice immunization, sample collection and challenge experiment

5-week-old female BALB/c mice were purchased from Labanimal (Seoul, South Korea). Mice were given sterile food and fresh water *ad libitum* throughout the study, and were kept under pathogen-free conditions in controlled-environment isolation rooms (24°C ± 2°C, 12 h light/dark cycle and 40-70% relative humidity). The mice were initially allowed to acclimatize to the laboratory environment for 1 week before experimentation began. The mice were orally administered 1 × 10^9^ CFU (colony forming unit) of *S* *. Typhimurium* strains at 2 weeks of age and orally boosted at 4 weeks of age. The mice were observed at least once daily for general appearance, a variety of neurologic and behavioral change, clinical signs. The group A mice were immunized with PBS as a control, the group B mice received BRD509, the group C mice received BRD509 *fljB* ^+^ *fliC* ^+^ and the group D mice were immunized and boosted with BRD509 *fljB* ^+^ *fliC* ^*+*^. For blood sampling, mice were anesthetized in diethyl ether and blood samples were collected by retro-orbital puncture with a Pasteur pipette at 0, 2, and 4 weeks post-priming immunization (PPI) to evaluate the serum antibody responses. The serum samples were separated by centrifugation at 3,000*g* for 5 min. The virulent wild-type SL1344 strain cultures were harvested by centrifugation and were diluted to approximately 1 × 10^8^ or 3 × 10^9^ CFU in 20 µl of sterile PBS. All mice were challenged orally on week 4 PPI with the diluted virulent strain SL1344. At 14 days after the challenge, all surviving mice were sacrificed by cervical dislocation and the liver and splenic tissues were aseptically harvested. The harvested spleens and livers were homogenized in PBS and plated on LB agar for CFU determination, and then the challenge strain was isolated after incubation at 37°C for 24 h [22].

### Immune response measurements

The serum IgG antibody responses against flagella or lipopolysaccharide (LPS) were investigated by enzyme-linked immunosorbent assay (ELISA) as previously described [22], with some modification. Briefly, 96-well ELISA plates (Nunc, Roskilde, Denmark) were coated with 100 µl of *S* *. Typhimurium* flagellin or LPS (Calbiochem, CA, USA) diluted in carbonate buffer (5 µg/ml) at 4°C overnight. *S* *. Typhimurium* flagellin was isolated from BRD 509 *fljB* ^+^ *fliC* ^*+*^ as reported by Ayse Nalbantsoy, et al. [24]. The plates were washed three times with PBS containing 0.05% Tween-20 (PBST) and blocked with 3% BSA (in PBS) for 2 h at 37°C. Sera obtained from immunized mice were serially diluted tenfold with 3% BSA and added to the wells in triplicates and incubated for 1 h at 37°C for examination of the IgG titers. The plates were treated with HRP-conjugated goat anti-mouse IgG (1:3,000; Bio-Rad) and incubated for 1 h at 37°C. After five times washing with PBST, the enzymatic reactions were developed with 3,3´,5,5´–tetramethylbenzidine substrate solution (BD Biosciences, Franklin Lakes, NJ, USA) for 30 min. The reaction was stopped using 2N H_2_SO_4_ and the absorbance was measured by an automated ELISA spectrophotometer (Bio-Rad) at 450 nm. The standard curve of IgG is prepared from the concentration of standard solutions and their absorbance. The concentration of antibody for each sample were calculated from the standard curve and multiplied by the dilution factor and was expressed as the number of ng/ml. Titers were calculated by interpolation in a standard curve as the inverse of the dilution that produces an absorbance value of 0.2 above the blank (ELISA units/ml).

### Statistical analysis

Statistically significant differences between the strains were determined by the *P* values of Student’s *t*-test. Differences were considered statistically significant at a *P* value of < 0.05.

## Results

### Production of both FliC and FljB flagellar filaments at the same time in the *iacP* mutant strain

**Figure 1:**
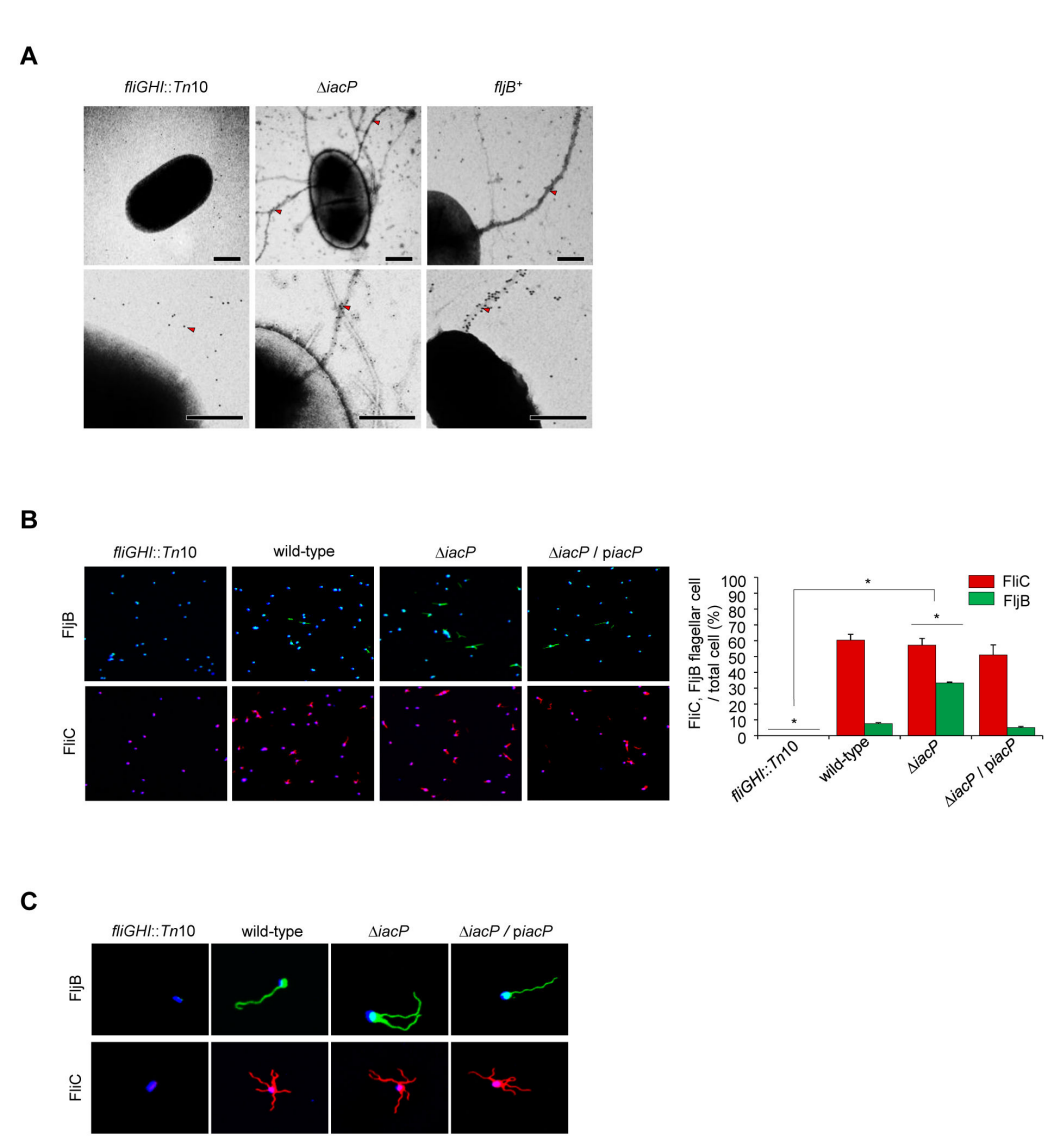
The *iacP* mutant strain has two different types of flagellar filaments. (A) The bacteria cell surface was immunogold-labeled and negatively stained for transmission electron microscopy. The bacteria were deposited on the grid and incubated with an anti-FljB primary antibody; this was followed by incubation with protein G–gold and negative staining with uranyl acetate. The images at the bottom panel show higher magnification of electron micrographs. The 10-nm gold particles (arrows) decorate the flagella structures. The images are representative of three independent experiments. Scale bars, 0.5 µm. (B) The flagellar filaments were stained with FITC-conjugated anti-FljB antibody and rhodamine-conjugated anti-FliC antibody. The nucleus and bacteria were stained with Hoechst 33342 dye (original magnification, × 630) (Right panel). For each strain, the number of flagella per cell was quantified from at least 50 cells. Error bars indicate the mean ± SD (standard deviation) of three independent experiments. Asterisks indicate statistically significant difference from the control group, determined by a Student’s *t* test (*P* < 0.05). (C) To visualize the flagellar filaments on a single bacterium, the images in B panel show magnified views of at least 100 cells.

As the *iacP* mutant strain could significantly induce the expression of *fljB* [7], we confirmed the formation of FljB-containing flagella in the *iacP* mutant strain by performing immunogold labeling transmission electron microscopy and immunofluorescence detection. As shown in Figure 1A, FljB-specific gold particles were mainly observed along a portion of flagella on the surface of the *iacP* mutant, although the nonspecific background staining was detected basally. To reduce nonspecific background staining, gold-labeled secondary antibody used at increasing dilution and images were visualized at a higher magnification (Figure 1A bottom panel). Unlabeled flagella filaments in the *iacP* mutant strain are thought to be composed of FliC flagellin. Strain expressing only FljB and a non-flagellated *fliGHI*::Tn*10* strain were used as controls (Figure 1A). Consistent with the results in Figure 1A, both FljB- and FliC-expressing flagella were observed in the *iacP* mutant by immunofluorescence with specific antibodies to FliC or FljB (Figure 1B and 1C). Flagella filament FljB proteins were easily detected in the *iacP* mutant culture, whereas the percentage of FljB-expressing cells were markedly decreased in wild-type and the *iacP* complementing strains. However, we observed no significant differences of FliC-expressing flagella in the wild-type strain, the *iacP* mutant strain and the *iacP* complementation strain. The *fliGHI*::Tn*10* mutant strain was used as a nonmotile control (Figure 1B). The larger magnification image shown in Figure 1C indicates that the FljB flagellar filaments are longer than the FliC flagellar filaments on a single bacterium. These results support the hypothesis that the *iacP* mutant strain simultaneously synthesizes two antigenically distinct flagellar filaments encoded by the flagellin genes, *fljB* and *fliC* [7].

### Confirmation of the expression of two flagellins in the *iacP* mutant strain

**Figure 2:**
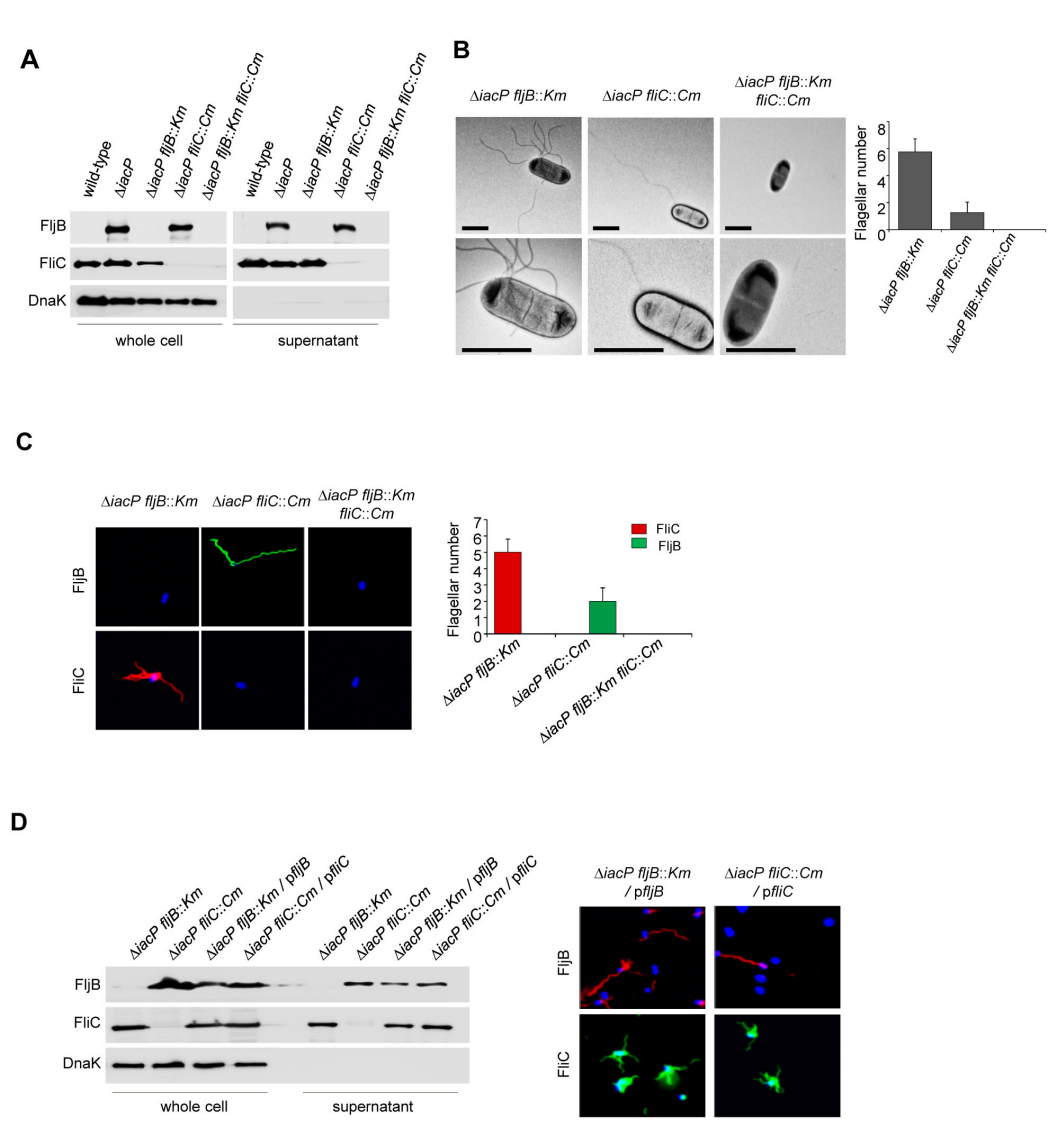
Flagella associated with *fljB* were observed in the *iacP fliC* double mutant. (A) The levels of FljB and FliC in the pellet and culture supernatants from all strains were determined by Western blotting with anti-FljB and anti-FliC antibodies. An anti-DnaK antibody was used as a loading control for cytoplasmic proteins. (B) A transmission electron micrograph of double mutant *iacP* and each *fljB*, *fliC* and triple mutant *iacP* *fljB* *fliC* was negatively stained with uranyl acetate. A higher magnification images is shown in the bottom panels (Right panel). The average number of flagella was determined from electron micrographs of at least 50 cells per strain. Representative images of three independent experiments are shown. The error bars represent ± SD of the mean. Scale bars, 2µm. (C) The flagellar filaments were visualized in different strains after immunostaining (as in Figure 1B and 1C). Bacterial DNA was stained with the DAPI (Right panel). Quantification of the flagella numbers of all strains in single bacterial cells. The error bars correspond to means ± SD. (D) Complementation of the *fljB* or the *fliC* genes in double mutant strains was examined by Western blotting and immunostaining. An anti-DnaK antibody was used as a control for cytoplasmic proteins (Right panel). To visualize flagellar filaments at the bacterial cells, the *iacP* *fljB*, the *iacP* *fliC* double mutant strain carrying the *fljB* or *fliC* gene on a plasmid were stained as described in Figures 1 and 2. The bacteria were stained with Hoechst 33342 dye.

To confirm the flagella expression in the *iacP* mutant strain, the *fliC*, *fljB* or both genes were disrupted in the *iacP* mutant strain, and then whole cell lysates and secreted proteins were prepared from the cultures of the wild-type strain, the *iacP* mutant strain, the *iacP fljB*, *iacP fliC* double mutant strain, and the *iacP fljB fliC* triple mutant strain. As shown in Figure 2A, we found that the flagella in the mutant strains were properly manufactured and the expression of FljB was observed in the *iacP fliC* double mutant strain similar to the *iacP* mutant strain. Interestingly, the confocal images in Figure 1 showed that the flagella consisting of flagellin FljB seemed to be fewer in number, but they were longer than the FliC flagella. To further characterize FliC and FljB filaments in the *iacP* mutant, flagellar filaments were observed in individual mutant strains based on immunofluorescence and electron microscopy. As shown in Figure 2B, the flagella filaments in the *iacP fliC* double mutant strain were significantly longer than the filaments in the *iacP fljB* double mutant, and the number of FljB flagella was less than that of FliC flagella. No flagellum was observed in the *iacP fljB fliC* triple mutant strain. Immunofluorescence analyses showed that the number of FliC flagella was approximately five to six, and the FljB-containing strain possessed one to three long flagella (Figure 2C), indicating that the biosynthetic process of FljB flagella may be different from that of FliC flagella and that flagellin FljB and FliC were not co-polymerized during flagellar synthesis. We next performed complementation studies with the *fljB*, *fliC*-encoding plasmids (P_BAD_-*fljB*, P_BAD_-*fliC*), in which the FljB and FliC productions were controlled by the arabinose-inducible promoter. Figure 2D shows that FljB and FliC were detected in the *iacP fljB* and *iacP fliC* double mutant strains by arabinose-inducible complementation, and the few long flagella that were present were composed of FljB flagellin. These results demonstrate that FljB expression was induced in the *iacP* mutant strain and that both types of flagella on a single bacterial cell surface were independently produced in the *iacP* mutant.

### Activation of NF-κB in bone marrow-derived macrophage (BMDM) and RAW 264.7 cells infected with the *iacP* mutant strain

**Figure 3:**
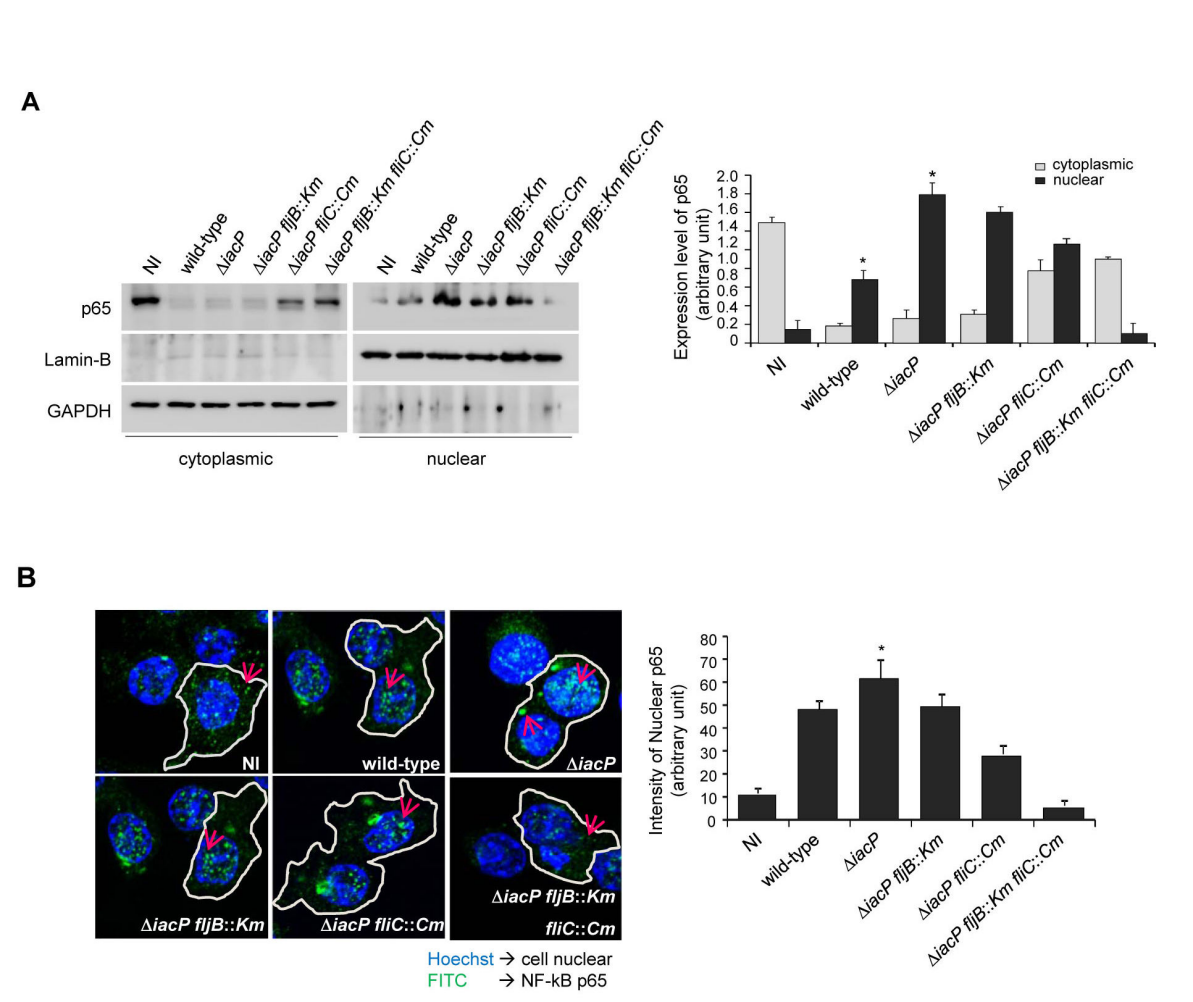
NF-κB activation is increased in Raw 264.7 cells infected with the *iacP* mutant. (A) The expression of NF-κB p65 was determined using immunoblot analysis with an anti-NF-κB p65 antibody in the cytoplasmic and nuclear fractions of *Salmonella* -infected Raw 264.7 cells. The anti-GAPDH and anti-Lamin B antibodies were used as cytoplasmic and nuclear protein markers, respectively (Right panel). Quantification of the amount of nuclear and cytoplasmic p65 expression levels in Raw 264.7 macrophages. Bars correspond to means ± SD. (B) The nuclear localization of NF-κB p65 as monitored by indirect immunofluorescence was used as readout for NF-κB activation. The cell nuclei were stained with DAPI, and p65 was visualized with FITC-conjugated anti-p65 antibody (Right panel). Quantification of nuclear p65 activation in Raw 264.7 cells was performed by densitometry analysis. Images from five different confocal planes (at least 30 cells) per sample were analyzed to determine the quantification of nuclear NF-κB p65. The error bars represent the means ± SD for three individual experiments. Analysis by Student’s *t* test indicated that the differences were statistically significant (*, *P* < 0.05).

The *S* *. Typhimurium* flagellin monomer has been demonstrated to induce the transcription of proinflammatory genes by activating NF-κB signaling via TLR5 [18,23]. In addition, after entry of *Salmonella* into host cells, monomeric flagellins are continuously translocated into the host cytosol by the virulence-associated T3SS [25,26] and are then recognized by a Nod-like receptor (NLR), which represents a key component of the host innate immune response [27,28]. Previous studies suggest that activation of NF-κB-dependent gene expression and interleukin 1β (IL-1β) secretion through the TLR5- and Ipaf-dependent signaling pathways was activated by *Salmonella* expressing two flagellin proteins, FliC (encoding phase 1 flagellin) and FljB (encoding phase 2 flagellin) [29]. To test whether the *iacP* mutant strain expressing FljB and FliC can activate the host innate immune response in a NF-κB-dependent manner, we investigated nuclear translocation of NF-κB in RAW 264.7 macrophages [21]. RAW 264.7 cells were infected with the wild-type *S* *. Typhimurium* , the *iacP* mutant and its isogenic mutant strains deficient in either one or both flagellin genes (*iacP fljB*, *iacP fliC* and *iacP fljB fliC* mutant strains). As shown in Figure 3A, infection with the wild-type strain resulted in increase of nuclear localization of the NF-κB p65 subunit, and NF-κB activation was markedly higher in the *iacP* mutant-infected cells. Infection with the *iacP fljB* mutant strains showed lower activation of NF-κB comparable to the level in wild-type Salmonella, and the *iacP fliC* mutant strains also presented significantly decreased levels of NF-κB-dependent gene activation. Treatment of RAW 264.7 cells with non-infected (NI) *Salmonella* did not affect activation of NF-κB. The observed nuclear localization of NF-κB and p65 was not due to differential bacterial uptake by RAW 264.7 macrophages (data not shown). Consistent with the immunoblot analyses, the frequency of NF-κB nuclear translocation quantified by confocal microscopy was the highest in the *iacP* mutant-infected macrophages (Figure 3B). These results indicate that the *iacP* mutant strain that produced both types of flagella could elicit strong flagellin-dependent NF-κB activation in RAW 264.7 macrophages.

**Figure 4:**
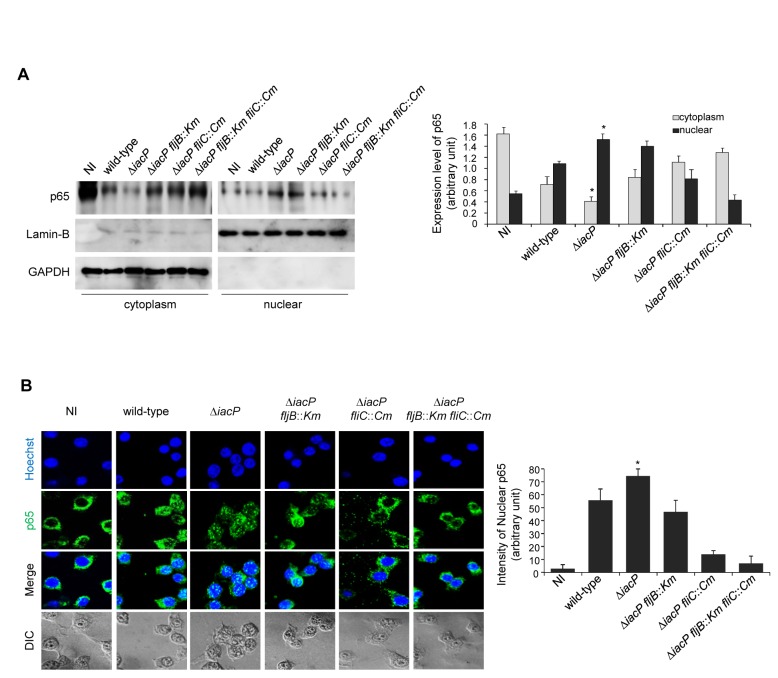
The *iacP* mutant induced the nuclear translocation of NF-κB in BMDM. (A) The expression levels of NF-κB p65 in the cytoplasmic and nuclear fractions were determined using immunoblot analysis with an anti-NF-κB p65 antibody. The data were normalized relative to Lamin B (nuclear protein) or GAPDH (cytoplasmic protein) as a protein loading control (Right panel). The expression levels of p65 in the nuclear and cytoplasmic fractions were quantified by densitometry analysis. Each band in blots was densitometrically normalized to GAPDH and Lamin B, and the levels of p65 in cytosolic and nuclear fractions are expressed as the mean ± SD from three separate experiments. (B) Nuclear localization of NF-κB p65 was detected by immunofluorescence staining in primary cultures of BMDMs. DNA in the cell nucleus was visualized by DAPI staining, and p65 was stained with a FITC-conjugated anti-p65 antibody (Right panel). NF-κB p65 nuclear activation was quantified by densitometry analysis. The data shown are representative of at least three independent experiments. The error bars correspond to the means ± SD. *, *P* < 0.05 (statistically significant difference from the control group).

Bone marrow-derived cells are thought to be important mediators of innate responses and mouse BMDM responds to bacterial flagellin in a MyD88-dependent manner, thereby inducing the production of proinflammatory cytokines [20]. We therefore used murine BMDM cultures to check the effect of an *iacP* mutant infection upon NF-κB activation. Consistent with the result of Figure 3, confocal immunofluorescence microscopy and Western blot analysis showed that nuclear localized NF-κB was elevated in the *iacP* mutant-infected cells (Figure 4A and 4B). Together, these results suggest that FliC and FljB produced by the *iacP* mutant strain may promote the activation of the NF-κB pathway in a p65-dependent manner, although differences in nuclear translocation of p65 among strains were seemingly minimal.

### Induction of proinflammatory cytokines by the *iacP* mutant strain in macrophage cells

**Figure 5:**
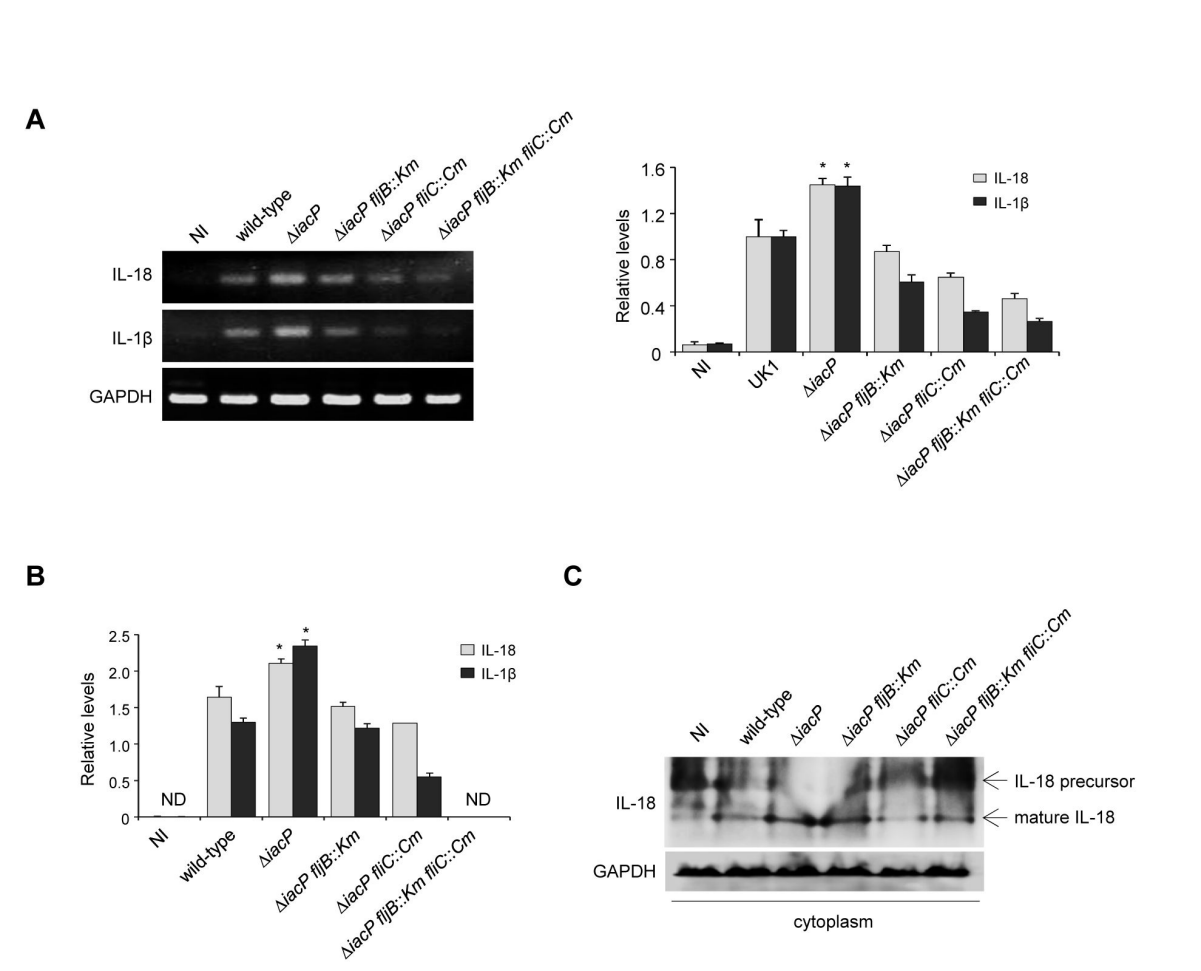
The *iacP* mutant enhanced cytokine-induced nuclear translocation of NF-κB in BMDM. The mRNA expression levels for the proinflammatory genes IL-18 and IL-1β were determined by reverse transcription-PCR (RT-PCR) experiment (A) and quantitative real-time PCR (qPCR) analysis (B). Band intensities of RT-PCR were quantified by densitometry and normalized to the level of the GAPDH. For the qPCR data, the expression values were normalized to the GAPDH level and reported relative to the expression level in the wild-type strain. The experiments were performed on three individual samples, and the representative data are shown. The error bars indicated the means ± SD. Analysis by Student’s *t* test indicated that the differences were statistically significant (*, *P* < 0.05). (C) BMDM infected with *S* *. Typhimurium* strains were analyzed at the protein level by Western blot analysis. The expression of IL-18 as a proinflammatory gene was determined by immunoblot analysis with anti-IL-18 antibody in the cytoplasmic fraction. An anti-GAPDH antibody was used as a loading control for cytoplasmic proteins. Representative data from three independent experiments are shown.

Macrophage cells are considered to be important in *Salmonella* infection, as they are known to produce a variety of proinflammatory cytokines that contribute to the protective host immune responses against *Salmonella* infection [30]. In addition, flagellin from *Salmonella* stimulates the production of chemokines and proinflammatory cytokines in host cells, including IL-18, IL-1β, IL-8, TNF-α and IL-6 [31]. Therefore, we tested whether the infection of the *iacP* mutant strain expressing two flagellin subtypes (FljB and FliC) could induce the release of proinflammatory cytokines such as IL-18 and IL-1β in the RAW 264.7 macrophage cell line. As shown in Figure 5A, RT-PCR analysis showed that the transcription levels of IL-18 and IL-1β were slightly but significantly increased in BMDMs infected with the *iacP* mutant strain over those in the wild-type or the *iacP fljB* mutant-infected cells. The qPCR data also confirmed the RT-PCR results; the *iacP* mutant strain had increased production of IL-18 and IL-1β in BMDMs. In BMDMs with non-infected control (NI) and with the *iacP fljB fliC* mutant infection, the levels of cytokines IL-18 and IL-1β were not detectable (Figure 5B). We subsequently determined whether IL-18 expression levels in the *iacP* mutant strain were regulated at the protein level. Total protein extracts from the all *Salmonella* strain-infected BMDMs were prepared and measured by immunoblot assays with an anti-IL-18 antibody. GAPDH served as a loading control. Figure 5C shows that the *iacP* mutant strain exhibited a high level of activity of the mature IL-18 compared with the wild-type strain, suggesting that dual expression of FljB and FliC in the *iacP* mutant can generate a greater immune response in the host cell. These results demonstrate that overexpressing flagellin through the *iacP* mutation may induce an immune response characterized by induction of the proinflammatory cytokines IL-18 and IL-1β, although differences in proinflammatory cytokines among strains were seemingly minimal response.

### Construction and characterization of an attenuated *Salmonella enterica* serovar Typhimurium strain expressing both FliC and FljB flagellins

We observed that NF-κB activation and proinflammatory cytokines IL-18 and IL-1β production in *Salmonella* -infected macrophages are dependent on the expression of FliC and FljB flagellins. Recent studies have reported that FljB has an adjuvant activity in the context of a broad range of recombinant *Salmonella* vaccine candidates and that the production of FljB results in attenuation of virulence in murine models of systemic infection [8,32]. Thus, we investigate whether as a vaccine candidate, the oral administration of *S* *. Typhimurium* simultaneously expressing FljB and FliC could enhance the immune response and confer protective immunity against a lethal challenge with a virulent strain in vaccinated mice.

**Figure 6:**
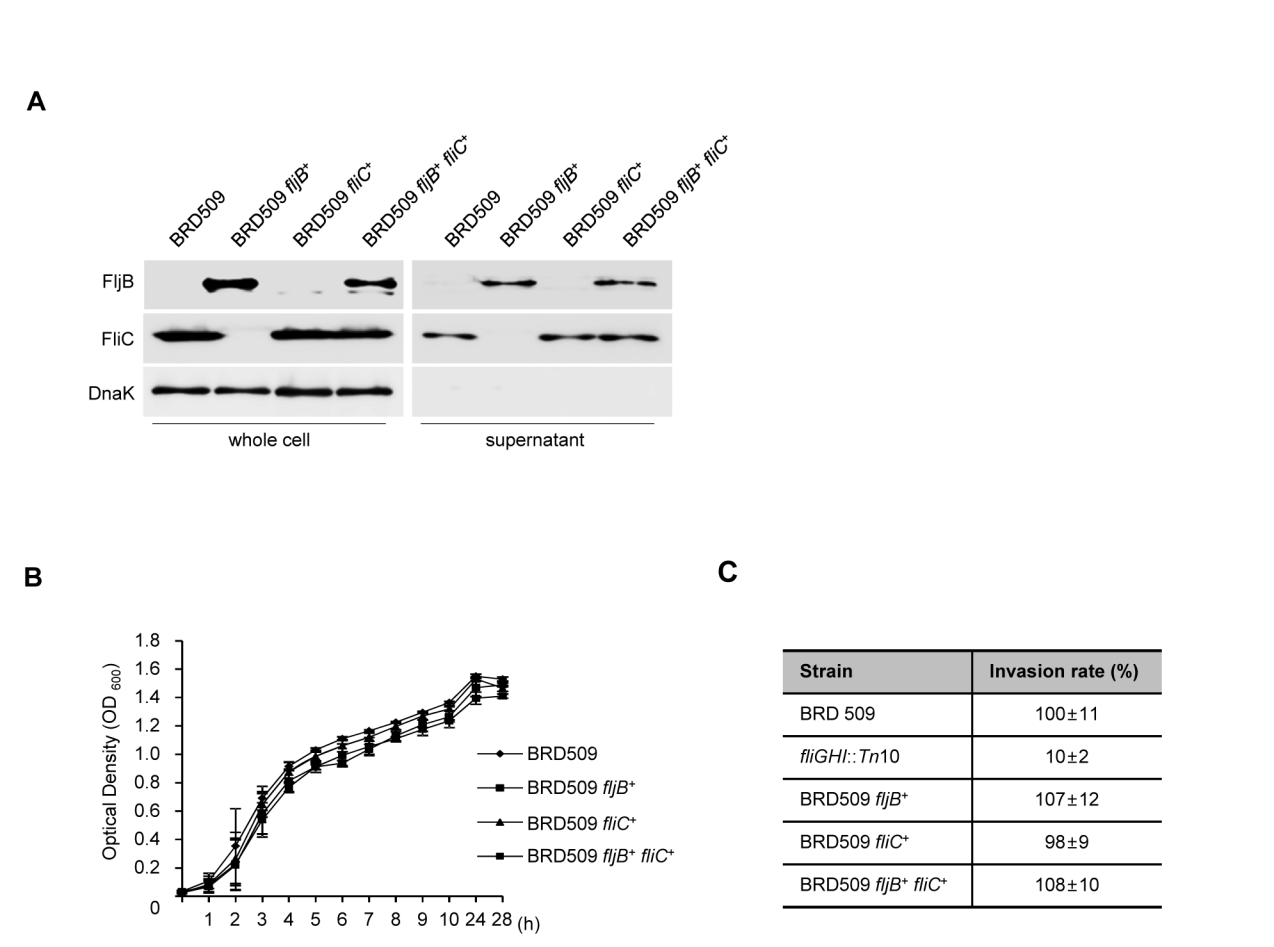
Construction and characterization of attenuated *S* *. Typhimurium* strain containing FliC and FljB. (A) The FljB levels of recombinant attenuated *S* *. typhimurium* *fljB* ^+^ *fliC* ^*+*^ strain that contains two different flagellin genes, *fljB* and *fliC*, in the pellet and culture supernatant were determined by Western blotting using anti-FljB and anti-FliC antibodies. An anti-DnaK antibody was used as a control for cytoplasmic proteins. (B) The growth curves of the recombinant bacteria strains in LB broth containing 0.3 M NaCl at 37°C. Data are means for three independent experiments. (C) To determine the invasion rate, recombinant bacteria strains were allowed to infect INT-407 epithelial cells for 45 min, after which a gentamicin protection assay was conducted. The number of infected bacteria was confirmed by plating the serial dilutions, and the bacterial invasion rates were calculated as a percentage of wild-type, which was set as 100%. All of the experiments were performed at least three times, and representative data are shown. Error bars correspond to the means ± SD.

We evaluated the vaccine efficacy of the FljB- and FliC-expressing recombinant strain using the attenuated *S* *. Typhimurium* strain BRD509 because the *iacP* mutant is less invasive than wild-type *S* *. Typhimurium* in INT-407 intestinal epithelial cells. The strains BRD509 *fljB* ^+^, BRD509 *fliC* ^+^, BRD509 *fljB* ^+^ *fliC* ^+^ were constructed from BRD509 by the disruption of *hin* recombinase, which is responsible for flagellar phase variation, as described previously [7]. To assess the expression levels of FljB and FliC in the construct strains, Western blot analysis was performed. As shown in Figure 6A, FliC production or secretion was detected in whole cell lysates and the supernatant fraction of the BRD509 and BRD509 *fliC* ^*+*^ strains, whereas the BRD509 *fljB* ^*+*^ strain produced only FljB flagellin. It was observed that the levels of production and secretion of the two flagellar proteins (FliC and FljB) in the BRD509 *fljB* ^+^ *fliC* ^+^ strain were similar to those of the BRD509 strain possessing one type of flagellin. The growth rate of these strains was not significantly different (Figure 6B), and Figure 6C shows that the host cell invasion rates of the four attenuated strains, BRD509, BRD509 *fljB* ^+^, BRD509 *fliC* ^+^ and BRD509 *fljB* ^+^ *fliC* ^+^ were similar. The non-motile strain *fliGHI*::Tn*10* was used as a negative control in the invasion assay. Hence, this result suggests that concomitant expression of FljB and FliC may not affect bacterial growth and the host cell invasion ability.

### Protection against a wild-type Salmonella challenge and the immune response to LPS and flagellin in mice orally immunized with attenuated *Salmonella* expressing FljB and FliC

**Figure 7:**
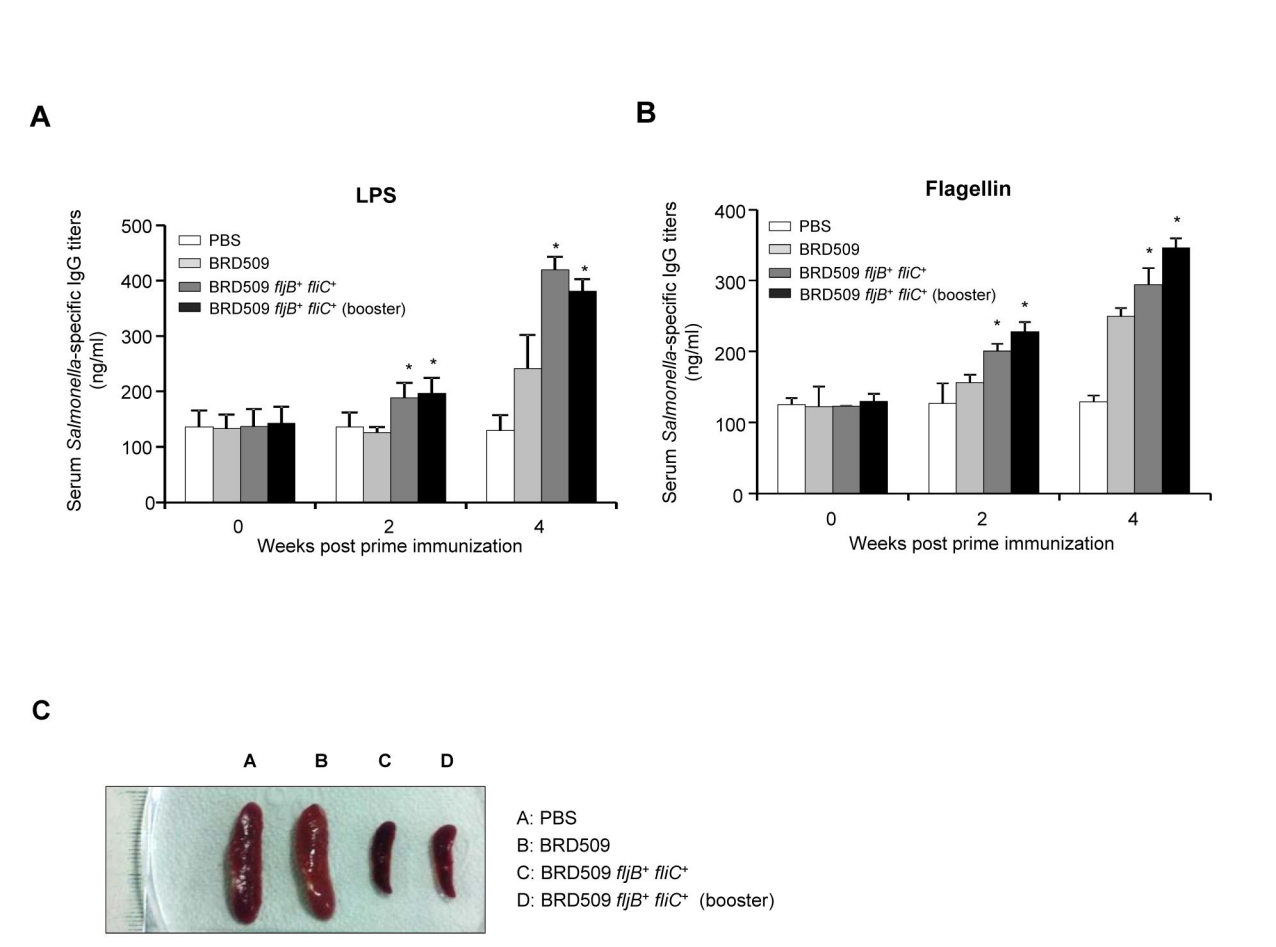
Immune responses to LPS and flagellin in mice orally inoculated with the FljB adjuvant strain. (A, B) IgG activity analysis of sera from *S* *. Typhimurium* -immunized mice at weeks 0, 2 and 4. The mice were immunized orally with 10^9^ CFU of BRD509, BRD *fljB* ^+^ *fliC* ^*+*^ and boosted with BRD *fljB* ^+^ *fliC* ^*+*^. A group inoculated with PBS was used as a control. The data shown are the values for all mice in each group, and the error bars indicate the SD. *, *P* < 0.05 (statistically significant difference from the control group). (C) Photographic images of isolated splenic tissue from the surviving mice after the challenge.

To examine the host immune response, 5-week-old female BALB/c mice were orally administered PBS (group A), BRD509 (group B), BRD509 *fljB* ^+^ *fliC* ^+^ (group C) and BRD509 *fljB* ^+^ *fliC* ^+^ with an additional booster immunization (group D). We measured the LPS-specific IgG and flagellin-specific IgG titers in mice serum collected at 0, 2 and 4 weeks post-inoculation by ELISA. The LPS-specific serum IgG titers of groups C and D were higher by approximately 3.2-fold and 3.0-fold, respectively, when compared with those of the control group A at 4 weeks post-inoculation. In mice immunized with BRD509 (group B), the LPS-specific serum IgG titers was lower than those in the serum of BRD509 *fljB* ^+^ *fliC* ^+^-immunized mice (groups C and D), however the titer was approximately 1.9-fold higher than that that of the control group A (PBS) at 4 weeks post-inoculation (Figure 7A). As shown in Figure 7B, the flagellin-specific serum IgG levels were increased approximately 2.3-fold and 2.7-fold in groups C and group D compared with the PBS-treated control at 4 weeks post-inoculation. The flagellin-specific serum IgG titer of mice in group B was also significantly 2.0-fold higher than the titer of mice of group A at 4 weeks post-inoculation. These results suggest that the dual expression of FliC and FljB enhanced the serum IgG responses to *Salmonella* LPS and flagellin.

The immunized mice were orally challenged with 1 × 10^8^ and 3 × 10^9^ CFU of the virulent *S* *. Typhimurium* SL1344 strain at 4 weeks post-inoculation, respectively. As shown in Table 3, none of the control mice (group A) challenged with the virulent SL1344 strain (3 × 10^9^ CFU) survived at day 14, whereas 40% of the BRD509 (group B)-immunized mice, 80% of the BRD509 *fljB* ^+^ *fliC* ^+^ (group C)-immunized mice and 100% of the boosted BRD509 *fljB* ^+^ *fliC* ^+^ (group D)-immunized mice survived until day 14 after challenge. At 14 days after the challenge, the spleen and liver tissues from the individual surviving mice were isolated, and the bacterial loads (CFU/g) were measured (Figure 7C, Table 3). The tissues of the infected mice were found to have over 200 colonies of bacteria in the total tissues. The numbers of bacteria in the spleen and liver were significantly lower in group D than in the other groups challenged with 3 × 10^9^ CFU of the virulent SL1344 strain (Table 3). Moreover, the spleens of the group C and D mice were smaller compared with those of group A and B mice (Figure 7C). These results show that the BRD509 *fljB* ^+^ *fliC* ^+^ strains conferred the best protection against virulent *Salmonella* SL1344 infection.

**Table 3:** Immunization, isolation of the challenge strains from organs and the mortality after challenge^**a**^.

Group	Immunization		Challenge (1 × 10^8^)				Challenge (3 × 10^9^)			
	Strain	Booster	No. of mice used	Mortality (%)	No. of infected spleens/no.of spleens tested	No. of infected livers/no.of livers tested	No. of mice used	Mortality (%)	No. of infected spleens/no.of spleens tested	No. of infected livers/no.of livers tested
A	PBS	None	5	80	1/1	1/1	5	100	0/0	0/0
B	BRD509	None	5	40	2/3	3/3	5	60	1/2	2/2
C	BRD509 *fljB* ^+^ *fliC* ^+^	None	5	0	1/5	2/5	5	20	1/4	4/4
D	BRD509 *fljB* ^+^ *fliC* ^+^	BRD509 *fljB* ^+^ *fliC* ^+^	5	0	1/5	1/5	5	0	1/5	2/5

^a^Mice were immunized orally with 1 × 10^9^ CFU in a volume of 20 µl, with 2 weeks immunization and 4 weeks booster vaccination and orally challenged 4 weeks later with 1 × 10^8^ or 3 × 10^9^ CFU of virulent strain (SL1344), respectively. Sera were collected by retro-orbital puncture with a Pasteur pipette at 0, 2, and 4 weeks post-priming immunization (PPI). The mice were monitored daily for 14 days.

## Discussion

IacP, an invasion-associated acyl carrier protein, acts as a *S* *. Typhimurium* virulence factor by promoting the transport of SPI-1 type III effector proteins during host cell entry [7,33]. In addition, the disruption of the *iacP* gene produces two different flagellin proteins, FliC (phase 1) and FljB (phase 2), which result in higher flagellar numbers per cell than the wild-type strain [7]. In this study, we showed that the *iacP* mutant strain producing two different flagellar filament proteins FliC and FljB induces immune responses by enhancing NF-κB activation and proinflammatory cytokine expression in the host cells.

The expression of two antigenically distinct flagellin genes, *fliC* and *fljB*, in the *iacP* mutant strain was confirmed by immunogold electron microscopy and immunofluorescence assays. Bacterial flagellin, the structural component of flagellar filament, is usually assembled in the flagellum, accelerating the bacterial invasion of the host cells, conferring a survival advantage during early infection in mice and activating the host innate immune response [22]. The importance of flagellin-induced gene expression has been acknowledged for *Salmonella* infection because flagella expression provides a survival advantage in the early stage of infection. The synthesis of flagella is then turned off to minimize host recognition once infection has been established [34]. Hence, the regulation of the *Salmonella* flagellar filament is critical for the interactions between the host cell and the *Salmonella* pathogen*.*

However, the wild-type strain and *iacP* mutant possessing the *iacP*-complementation plasmid mostly have FliC flagellin, whereas the *iacP* mutant produces one or two long flagellar filaments. These results indicate that FljB is responsible for the production of more flagellar filaments in the *iacP* mutant strain. This study found that the phase 2 flagella consisted of FljB subunits that appeared to be longer than the phase 1 flagella (FliC), and up to three flagella were assembled at the bacterial cell surface. Upon bacterial adhesion to the host surface, materials can be rapidly transported along the length of the flagellum. Therefore, the filament length and number are important for the assembly of the flagella, motility and signal transduction [35]. In addition, long filaments are clearly more capable than short filaments of pulling or pushing the cell body toward a new direction and are the major factors leading to the immune response [36]. If the total amount of FliC or FljB flagellar subunits that were encoded from pBAD24 plasmids were similarly produced by arabinose induction, it appeared that the FljB monomers were polymerized onto fewer (1 to 3) phase 2 flagella than phase 1 flagella (5 to 7), thereby increasing the flagellar length. However, it remains unknown whether FljB was specifically targeted to the flagellar component or whether fewer phase 2 flagella than phase 1 flagella were activated for the polymerization of FljB monomers.

Recent studies show that the flagellin components FliC and/or FljB of *S* *. Typhimurium* are capable of activating the innate immune system via specific interactions with TLR5 and NLR that lead to immune protection against *Salmonella* infection. *Salmonella* overexpressing recombinant flagellin was highly attenuated by activation of the innate immune response to flagellin *in vivo* [27]. We found that NF-κB-dependent gene expression and cytokine production in macrophages were robustly stimulated by infection with *Salmonella* expressing two flagellin genes, *fliC* and *fljB*, in comparison with cell strains expressing only one type of flagellar antigen. Thus, the flagella overproduction in the *iacP* mutant strain could have elicited a greater host innate immune response and may have contributed to the attenuation of virulence in *Salmonella* pathogenesis.

Therefore, the lengths and numbers of flagellar filaments may play a significant role in determining the virulence of pathogenic bacteria and activation of the immune responses in host cells, which results in virulence attenuation and induction of a robust immune response in the *iacP* mutant strain with longer flagella present in greater numbers. Further studies are necessary to determine the morphological features of flagella composed of two species of flagellin (FliC, FljB) and compare them with FliC or FljB monophasic *Salmonella* .

Although many reports suggest the usage of the FljB protein as an immune-stimulant in the vaccine strategy, the effect of the combination of FliC and FljB co-expressed in a single cell has not been tested. To examine the vaccine adjuvant activity of the FljB flagellin protein in a mouse model, we constructed a recombinant attenuated *S* *. Typhimurium* strain containing the FljB and FliC flagellins and then performed immunization to determine the protective efficacy against an oral challenge with *Salmonella* . The data indicate that the FljB adjuvant strain can effectively induce a systemic immune response by stimulating serum IgG titers to the *Salmonella* -specific LPS and flagellin. Our results demonstrate that almost of all of the mice vaccinated with the strain expressing both flagellin genes (*fljB* and *fliC*) were protected against *Salmonellosis*.

In conclusion, we show that FljB increased the induction of the host immune response against virulent *Salmonella* . In recent years, an increasing number of studies demonstrated the effectiveness of membrane-anchored flagellin as a mucosal adjuvant, as well as its ability to promote cytokine expression by innate immune cells and trigger the generalized recruitment of lymphocytes (T and B cells) to secondary lymphoid organs [32]. The adaptability of flagellin has enabled the generation and immunization of a range of flagellin-based fusion proteins that have proven to be cost-effective antibacterial vaccines in animal models. Accordingly, vaccine studies of the adjuvant effect of flagellin may have important practical applications for current and future research.
